# Solidarity riots in the diffusion of collective action: Doing historical research to develop theory in social psychology

**DOI:** 10.1111/bjso.12870

**Published:** 2025-02-27

**Authors:** John Drury, Roger Ball, Steve Poole

**Affiliations:** ^1^ School of Psychology University of Sussex Brighton UK; ^2^ Faculty of Arts, Creative Industries, and Education University of the West of England Bristol UK

**Keywords:** 1831 reform movement, collective action, history, protest, riots, social identity

## Abstract

Both psychology and historical studies have addressed the question of the diffusion of collective action events, although using very different methodological approaches and with differing concepts. In the present paper, we present a novel approach, combining historiographical research methods with analytic concepts from social psychology, to explore the psychological processes underlying riot diffusion. Using archive data from the 1831 wave of ‘reform’ riots, thick description of two collective action events provides evidence that the purpose of participants' actions was to prevent troops from passing through their towns to put down riots elsewhere. Their actions to support rioters in another location involved risk to themselves, and so can't easily be explained in terms of personal or local self‐interest. Instead, the evidence—in the form of context, utterances, and observations—is more consistent with the idea of common identity between people in the different locations motivating pre‐emptive solidarity, inadvertently spreading the riots. The use of historical archive data and historiographical research methods, suggesting a previously undocumented form of solidarity between participants at riot events, contributes to new understandings of the diffusion of collective action and how to study it in both historical studies and social psychology.

## INTRODUCTION

A wave of protests or riots is an indication of significant social discord. Such waves can also have significant social impacts. The 2011 Arab Spring forced several rulers from office; in 2018, *gilets jaunes* protests and riots forced the French government to scrap a fuel tax (Willsher, 2018); and the 2020 Black Lives Matter protests led to changes in policing practices in the United States (Ray, 2012). Going back further, waves of riots in 18th‐century France (Rudé, 1964) and England (Thompson, 1971) forced the authorities to meet the subsistence needs of the poor, including concessions in the price of bread and other provisions. As well as their social importance, waves of collective action events like these matter for theory. Evidence that collective action events influence the likelihood of further collective action events might indicate that participants see themselves as part of a politically conscious social movement (Myers & Przybysz, 2010). It is important, therefore, to understand how such influence between events happens.

Both psychology and historical studies have addressed the question of the diffusion of collective action events, although using very different methodological approaches and with differing concepts. Historical studies have provided detailed narratives of the context, timeline, actions, and meanings of waves of riots, while social psychology has suggested some of the possible psychological processes behind such waves. Historical research uses materials (archive data, including court records, correspondence, and newspaper articles) and studies events that are normally excluded from the scope of social psychology research because of the length of time since they took place. However, we argue that because the techniques of historical research can provide evidence on participants' beliefs, perceptions, and motives in long‐past riot events, such research can contribute to contemporary debates in social psychology. Specifically, such research can help us to develop theory in relation to the process of spread between such events, something which existing theories have yet to address satisfactorily. In the present paper, we therefore present a novel integration of historiographic research methods and social psychological concepts, in the form of a survey of events and in‐depth case studies of two riots that took place in England as part of the 1831 wave of ‘reform’ riots.

### Understanding the diffusion of collective action events in history and other disciplines

Interdependence of collective action events—i.e., events influencing the likelihood of further such events—has been systematically investigated since the historian Rudé's (1964) cartographic work on the ‘Captain Swing’ agricultural riots of 1830. His analysis suggested that, in addition to the common grievances across the different locations, riots in one location influenced the likelihood of occurrence in further locations, eventually forming a wave across southern England. Further advances in understanding riot diffusion came with the advent of improved access to computers for mathematical modelling (Ball, 2012). Perhaps the most significant of these were made using a statistical approach called event history analysis. Thus, examining data on the US urban ‘race’ riots 1961 to 1968, Myers (1997) demonstrated that previous rioting was a significant predictor of the hazard of rioting in another city even after all other predictors (including grievances around wages and unemployment levels) had been included. Myers also showed that severe riots were significantly more influential than less intense incidents and that influence decayed rapidly over time.

How did this diffusion process happen? The term ‘contagion’ is often found in the writings of historians, sociologists, criminologists, and others when discussing riot diffusion (e.g., Baudains, Johnson, & Braithwaite, 2013; Kucharski, 2020; Midlarsky, 1978; Myers, 1997, 2000). However, most people ‘exposed’ to rioting do not join in (Reicher & Stott, 2011). Even for basic behavioural responses (such as emotions), there is evidence that prior beliefs shape and limit emulation (Parkinson, 2019); hence the mindlessness suggested by ‘contagion’ explanations (e.g., Le Bon, 1965) is incorrect.

Despite their frequent use of the term ‘contagion’, the mechanism that many historians and sociologists offer to explain riot diffusion is the opposite: conscious emulation via learning and inspiration based on costs‐benefits analysis (e.g., Aidt et al., 2022; Baudains, Braithwaite, & Johnson, 2013; Davies et al., 2013). As Myers and Przybysz (2010) put it, diffusion is a ‘rational form of inter‐actor influence. Potential actors observe and evaluate the outcomes of others’ behaviours, and then make a decision for themselves about whether or not to adopt the behaviour’ (p. 64).

A criticism made of this rational‐choice approach to collective action is that it relies on the concept of ‘self‐interest’ but it fails to theorize the self (Drury & Reicher, 2020). It does not explain how interests come to be shared, or how the same individual can have multiple and sometimes conflicting interests (in particular, personal versus collective self‐interests).

Importantly, moreover, rational‐choice explanations for the diffusion of collective action events are almost entirely speculative. Almost all of the recent research suggesting that influence between collective action events occurs through emulation via rational choice has examined only aggregate data, such as the occurrence of riot events (Myers, 1997), recorded crime data (e.g., Davies et al., 2013), or the nature and size of riots (Aidt et al., 2022). There is a need to examine the beliefs, perceptions, and motivations of participants in the locations influenced by previous collective action events. We argue that combining the historiographical research method of fine‐grained thick description (e.g., Bohstedt & Williams, 1988) with analytic concepts from social psychology can suggest new insights on the process of influence between collective action events. In effect, we propose a new kind of collaborative social psycho‐history approach for understanding the spread of collective action events. As far as we are aware, this is the first attempt of its kind.^1^

### Doing historical research to develop theory in social psychology

A recent series of studies of urban riots suggests that collective action events can spread via multiple processes (Drury et al., 2020, 2022). The 2011 English riots began in Tottenham, north London, 2 days after the fatal shooting by police of a local mixed‐heritage man, Mark Duggan. One process of influence between the events seemed to be via a common identity. Thus, some interviewees in Brixton, South London, were angered by the police killing of Mark Duggan in Tottenham because they saw a commonality with local incidents of injustice and their own historical position as Black people. There was evidence, therefore, that the Brixton riot was driven by some participants' sense of how their community should act (i.e., to punish the police) based on a common group membership with people in Tottenham.

Historical data and historiographical research methods offer a valuable opportunity to significantly develop existing work. They can both contribute substantively to developing further hypotheses about how collective action events spread and can also form the basis of a novel methodological approach to addressing social psychological questions. In terms of the substance, in the present paper we use historical data and historiographical research methods to explore whether there is evidence to suggest that rational‐choice, common identity, or other kinds of processes can help account for patterns of diffusion found in a series of riots. In terms of the new methodological approach, we suggest that historical events and archive data normally excluded from psychology research—including witness statements captured in contemporaneous newspaper reports, correspondence, and government reports from crowd events long in the past—can be analysed for evidence of participants' beliefs, motivations, and perceptions. This therefore offers an alternative to the usual kinds of designs and data that feature in collective action research today—i.e., analysis of interviews and videos (e.g., Drury et al., 2020; Reicher, 1984), posts on social media (Smith et al., 2015), and questionnaires (e.g., Tausch & Becker, 2013)—thereby extending the scope of social psychology research and raising the prospect of insights not possible with the usual methods and examples.

There are some precedents for our approach. Historical research has been important in the development of theories of crowd behaviour in psychology. Thompson's (1971) detailed study of the eighteenth‐century English food riots was crucial in showing exactly what is wrong with Le Bon's (1965) classical crowd psychology and in pointing to the need to explain the limits of behaviour in ‘violent’ crowd events (Drury & Reicher, 2020). More recently, historical events and archives have been used to develop theory in other domains of crowd behaviour (e.g., Barr et al., 2024). However, whereas previous social psychology has referred to or drawn on examples from historical analysis (e.g., Reicher, 1984), or used methods similar to historical thick description as a complement to an interview study (e.g., Drury et al., 2020), here we do something new, which is to make the historiographic analysis, informed by social psychological concepts, the main focus of the study.

The present study therefore offers both a historiographical analysis to develop a social psychological account of social influence between collective action events and a novel historiographical study of riot spread through the use of social psychological theory. Our overall research question is *how did collective action events spread between locations in the wave of ‘reform’ riots in October 1831?* Specifically, we explore whether there is any evidence of social influence between locations that saw rioting and, if so, what the process was in terms of beliefs, perceptions, and motivations.

### The present study

In the early 1830s in the UK, a wide range of progressive causes and aspirations were attached to the idea of ‘reform’, and this was the basis of a significant campaign in that name across the country. As Innes (2022, p. 224) puts it, ‘The word [reform] … connoted the rooting out of corruption and abuse… The favourite object of reform was Parliament: that is, the electoral system.’ Therefore, there was widespread hope invested in the Second Reform Bill (1831), which was seen as a test case of support for (or opposition to) progressive causes.

However, the Bill was rejected by the House of Lords, now the last bastion of traditional opposition to a widening of the franchise and interference with the constitution. Tory opponents of reform feared not only an erosion of their own political privileges but also the unleashing of an unstoppable process of progressive change, the final act of which would be the seizure of power by a working class that they considered unfit to govern.

Rioting and other collective action events began immediately after the Lords' rejection. From October to December 1831, a wave consisting of ~500 public meetings, peaceful protests, disturbances, and riots swept across Britain and Ireland in the name of ‘reform’.

However, in historical studies, the spread of the ‘reform riots’ across some parts of the UK remains largely unexplained. Why did they occur when and where they did? What were the connections between these locations and events, if any?

We carried out a series of case studies of events in the 1831 wave, focused on the previously neglected South‐West of England, alongside an overview of the nature and sequence of all events in the overall wave. Two other surveys of the period were already in existence (Horn & Tilly, 2009; Tiratelli, 2020), but they were limited either in geographical scope or in sources. Our survey enabled some prima facie analytic claims about the occurrence of interdependence and the underlying process.

In contrast to contemporary urban riots, rioters in the 1830s obviously cannot be interviewed but also left little record of their beliefs, perceptions, and motivations. For evidence, we are therefore reliant on whether witnesses recorded any utterances, material culture (such as banners, flags, posters, and leaflets), and contemporaneous observations of crowd objectives. Across the events we analysed, while interdependence could be inferred in some cases, evidence of the underlying process of social influence was less common. However, in two of the case studies—Bath and Newport—we found evidence of an apparent solidarity motive linking local participation to the riot in nearby Bristol. Therefore, after summarizing the survey, the analysis focuses on these two case studies.

## METHODS

### Overall survey of collective action events

#### Sources

The data for the overall survey came from two main sources. The first was newspapers (Times Digital Archive, ProQuest, British Newspaper Archive, and Welsh newspapers online at the National Library of Wales; a few newspapers that were not online were surveyed in Somerset Heritage Centre and Bath Records Office). The second source was correspondence with the Home Office, sent from local landowners or officials (mayors, aldermen, magistrates or their clerks) and giving reports on ‘disturbances’, available at The National Archives (Home Office: Counties Correspondence 1820–1850 Ref. HO 52/12–16, 1831). A three‐month time span (1 October to 31 December 1831) was selected for scoping purposes and then, after a significant amount of data was collected, confirmed as encompassing the wave of collective action events. Additionally, we drew on a dataset on locations of Political Unions given in LoPatin (1999, p. 182 n.57 and pp. 174–177).

The collection of data was carried out in three phases. First, a politically diverse selection of national newspapers was read in detail day by day for the three‐month period to isolate reform‐related collective action events. In the second phase, 149 regional newspapers were searched for terms relating to reform protest. In the third phase, correspondence from county authorities to the Home Secretary concerning public order was surveyed, along with lists of the locations of reform meetings published in particular newspapers and the dataset giving locations where political unions had formed in the period (LoPatin, 1999). This evidence was then used to inform targeted searches amongst the local and national press, making phases two and three of the search process iterative. Where possible, cross‐referencing between multiple sources was carried out to triangulate collective action events.

#### Analysis

The kinds of events that were relevant to the survey had to have a direct relationship to Parliamentary reform and involve collective action, whether peaceful or violent. We categorized reform‐related collective action events as either *protests* (non‐violent public events), *disturbances* (minor violent event, minor damage, without yeomanry or military intervention or significant casualties) or *riots* (major violent event with significant damage to property, injury or loss of life, mass participation, significant duration, use of the Riot Act and intervention of yeomanry or military). The types of data collected for each collective action event included spatial and temporal characteristics, descriptions of the size and composition of crowds, and the nature of the protest within an assigned typology.^2^ The collective action event data was then collated chronologically.

### Bath and Newport case studies

#### Sources

The primary sources for the two case studies were accessed from the following locations: Bath Record Office (newspapers, court papers, private correspondence); Newport Museum and Art Gallery Local Studies collection (poll books, civic data, borough inquiries); Somerset Heritage Centre (court papers, yeomanry records); The National Archive (Home Office and War Office correspondence and registers); British Newspaper Archive and Welsh Newspapers at the National Library of Wales (online newspapers); contemporary governmental reports (online); contemporary regimental histories of Yeomanry units; Victoria Art Gallery (contemporary images). In addition, biographical and prosopographical data on the targets of the rioters and arrestees was gathered from court records and online sources including parish records, poll books, city directories, census data, prison and convict registers, and transportation records.

#### Data triangulation

The triangulation of different sources to substantiate the timing, location, and content of a particular incident within a riot event used in the case studies is similar to that used to analyse events in the August 2011 wave of riots in England. (See for example Stott et al. (2018) and Ball et al. (2019)). In the present case, there are essentially three types of written information available on riots and disturbances in the late modern period^3^ that could be used in the triangulation: real‐time accounts written *during* the events; *post‐hoc* accounts by participants and eyewitnesses; and evidence of *physical* damage to property or violence to the person. The few real‐time accounts for the two case studies presented here consisted of correspondence from county magistrates, military, and yeomanry officers to the Home Office or War Office in London. Some detailed accounts in the local newspapers were also clearly written from notes made by reporters who directly observed the events. Contemporary post‐hoc accounts, which were far more common, appeared in official correspondence to central government, court documentation (witness depositions, prosecution briefs, and transcripts of court cases), newspaper reports, and in a military journal. Evidence of physical damage to property, and thus targeting, came from some of these sources but also more systematically from a claim for compensation.

Through cross‐referencing these types of evidence and making reasoned assessments of the quantity and quality of the sources, confidence can be determined in a particular incident having occurred and when and where it happened. Central to this process is the creation of a timeline of incidents within the overall event. Contemporary maps, in situ fieldwork, and research workshops with local historians, archivists, and curators provided further sources for the verification of the evidence. These combined sources were then used to provide the basis for detailed narrative accounts which added general contextual information on the governance, economy, politics, popular culture, and class demography of the city in the period and more specifically the movement for reform in each region. As both case studies in this paper were related to the exceptional riot in Bristol, specific study of the economic, political, and social links between the locales and Bristol was also undertaken.

Two further areas of investigation in the case studies were the targets of the rioters and the composition of the crowd. Biographical information was collected on the people or owners of properties that were attacked by the crowd using census, parish, and civic records, city directories, poll books, and newspaper searches. For the crowd composition, typically a more difficult research problem, a prosopographical approach was taken with data collected on arrestees' names, gender, and age from newspaper and court records. This basic information was then augmented by using census and parish records, city directories, newspaper searches, criminal registers, poll books, and transportation records to investigate the backgrounds of the participants. Finally, where possible, any familial, residential, political, and economic connections between the participants were documented. Collating this richer data on arrestees allowed many characteristics of the crowd members to be ascertained beyond merely age and gender.^4^ These included occupation, place of residence, marital status, family size, literacy, property ownership, regional migration, previous criminal history, post‐reform voting rights, and any evidence of social networks. Combining this information with more general research into housing, employment, sanitation, and health, local politics, and governance in each city provided the context required for a ‘thick description’ analytic approach.

#### Evidencing beliefs, perceptions, and motives

Unlike many social psychology studies of riots (e.g., Drury et al., 2020, 2022; Reicher, 1984), the present research was not able to make use of participant interviews to make analytic claims about participants' subjective states. Therefore, we used the ‘thick description’ approach and multiple sources to examine any evidence of relevant beliefs, perceptions and motives, as they occurred within the events, or in witnesses' accounts. Evidence for beliefs, perceptions, and motives was therefore ascertained from recorded comments, shouts, chants, or slogans employed by members of the crowd. This was complemented by analysing the material culture of protest, gathering details of any flyers, posters, banners, and flags displayed. In addition, motives and meanings could sometimes be inferred by what witnesses said about the crowd and by what participants did (or did not do) in the particular context (as in the Newport case study).

For evidence specifically of social influence processes between events, for each riot location examined we gathered any instances of participants (or witnesses) referring to the location(s) where rioting had previously occurred (i.e., Bristol). These instances were then analysed to examine the extent to which it appeared that participants saw the other event(s) or locations as relevant to their own location (in terms of either learning, emulation, sense of connection, sense of injustice, emotion or any other belief, perception, or motive).

Finally, having established evidence of what participants seemed to be trying to achieve and any subjective connections between the locations, to further examine the nature and extent of the connection between each of Bath and Newport and the ‘source’ riot in Bristol, we then examined the evidence of any objective social links. Thus, evidence of strong family and work connections between Bristol and the other two locations might account for aspects of the evidence of motivations, perceptions, and beliefs.

## ANALYSIS

In the first part of the analysis, we draw upon the survey to provide an overview of diffusion in the October 1831 reform riots. This enables us to make preliminary observations about whether events were independent or interdependent. In the second part, we provide a summary of the Bristol riot, which was significant for the riots in nearby Bath and Newport. Finally, we present a detailed case study for each of the latter.

### Overall survey: the diffusion of riots in October 1831

**FIGURE 1:**
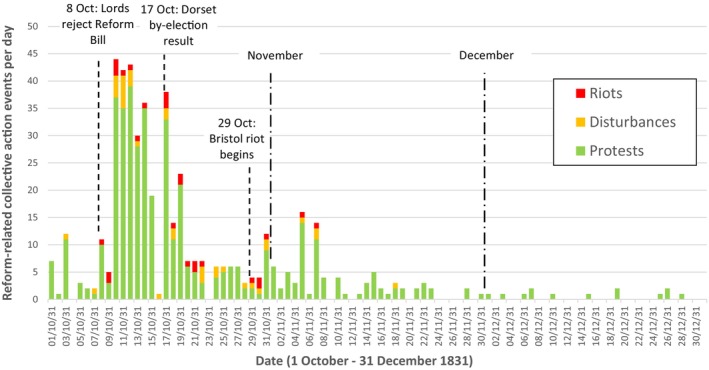
Reform‐related collective action events per day in Britain and Ireland, 1st October–31st December 1831.

**FIGURE 2:**
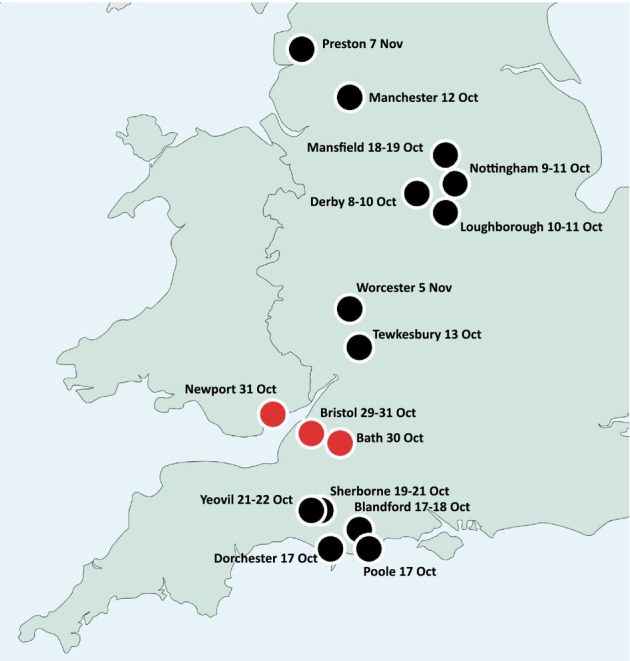
Locations and dates of reform‐related riots in England and Wales October–November 1831.

As Figure 1 and Figure 2 illustrate, there seem to be three temporal and spatial clusters of riots in October 1831. The first, situated in the east Midlands, relates to the riots in Derby and Nottingham which began on the evening of Saturday 8th and the morning of Sunday 9th of October respectively, and which each lasted for 3 days. These were each initiated by the arrival (by carriage) of news of the defeat of the Second Reform Bill. In each case, the riots were essentially attempts by the crowd to contest the result and punish the anti‐reformers and their authorities locally for the failure of the Bill (Ball et al., 2021). Therefore, these seem to be independent events, rather than events influenced by other riots.

The second cluster was in the South‐West of England, in Dorset and Somerset, over the period 17–22 October. This includes the riots in Dorchester, Blandford, Poole, Sherborne, and Yeovil. These incidents were all related to a high‐profile by‐election taking place in Dorset. In microcosm, the election represented the battle between what was widely described as ‘old corruption—i.e., those Tory MPs, peers, gentry, and clergy who supported the deeply flawed and exclusive electoral system—and the Whig party reformers. In a very close contest, the anti‐reform candidate Lord Ashley controversially won the seat in Dorchester on 17th October. Dorchester was the first place in the region to see serious collective violence, which originated at the by‐election hustings. The motive of the crowd here was again to punish anti‐reformers and their agents who were perceived to have corrupted the voting. Information on the contested result in Dorchester, as well as eyewitness accounts of attacks by yeomanry on the crowd, arrived the same evening in Blandford (Poole et al., 2023a) and then in Poole, which were the next places to riot (17–18 October). News of the contested election result and the riots would have been common knowledge in Sherborne the day after the election result (18 October). The Sherborne riots began the day after that, lasting for 3 days (19–21 October; Ball et al., 2023b). The riot in nearby Yeovil started on the third day of rioting in Sherborne and lasted two days (21–22 October; Poole et al., 2023b). Therefore, the people of Yeovil would have certainly heard about disorder in Sherborne and the others by that stage.

We infer from this sequence that there was a significant degree of interdependence in this second cluster. After the Dorchester riot, participants were responding to the rioting in other locations, not just to the victory of the anti‐reform candidate. However, in the data we have for these events, there is insufficient evidence to suggest the process: we were unable to locate data on perceptions, beliefs, or motives that related to the previous riot locations.

The third cluster was centred on the most serious riot of the period. Over 29–31 October, Bristol experienced severe and intense collective violence along with a bloody military intervention. Riots and disturbances also occurred in nearby Bath (30–31 October) and Newport (31 October). Here, we found evidence of social influence from Bristol to the other two locations in the form of recorded utterances and observations by witnesses. This is the basis of the case study analyses below.

### The Bristol riot^5^

The incident that led to the start of rioting in Bristol on Saturday 29th October was the provocative entry into the city of an outspoken anti‐reform judge and MP. A mass protest against the visit escalated into a riot after violence by special constables^6^ against the crowd outside the Mansion House (the mayor's official residence), which caused numerous injuries. After the crowd retaliated and began to swell in numbers, many of the special constables abandoned their posts, apparently emboldening the rioters. Later that evening, a military unit opened fire on rioters, killing one man and injuring several others.

At dawn in Bristol on Sunday 30th October, the Mayor of Bristol, several aldermen, and a few exhausted special constables were barricaded in the upper floor of the Mansion House after the rioting the day before. Although the crowd outside was small, their numbers were growing once again, and they started throwing stones at the building. Sometime after 9.00 am, after escaping from the besieged Mansion House, the mayor began seeking support from yeomanry and military units in nearby towns to quell the rioters, who had also been attacking Bristol's gaols. It was the response to this call for yeomanry and military units, along with what it signified to people living in Bath and Newport, that shaped actions in these two nearby towns.

### The Bath riot^7^

The request from the Bristol mayor for military help arrived with the Mayor of Bath early in the afternoon, 30th October. At 3.20 pm, the commander of the Bath troop of the North Somerset Yeomanry Regiment, Captain Charles Wilkins, received instructions to ‘assemble his men and march them to the neighbourhood of Bristol for the preservation of the peace’.^8^ Wilkins made a quick decision to muster his troops in the ‘usual rendezvous’ of Queen Square in central Bath and began sending out orders to round up his men.^9^

About 2 hours after Wilkins received his call for assistance, William Hall, mayor's officer, warned Robert Tothill, another mayor's officer, to expect trouble because he believed local people would be opposed to the yeomanry assembling in Bath.^10^ The glow and rising smoke from the fires in Bristol had been visible from some parts of Bath since late afternoon, so it would have been clear to many that something was happening. The Mayor later said he knew that ‘a considerable mob’ from Bath had gathered ‘in the streets leading to Bristol to collect information respecting that city’, and the *London Courier's* correspondent at Bath reported crowds collecting at the bottom of Union Street ‘to await the arrival of the mail from Bristol, to learn the news thence’. By this means they learned that the Yeomanry were being mustered, making them ‘exasperated’, and ‘determined those gentlemen should not quit Bath’.^11^ As the mayor's officer would later tell the assize hearing, ‘It was known at Bath that the Mansion House in Bristol was on fire, and the gaol, and that the prisoners had been liberated’.^12^ The mood on the streets was confirmed by one of the city's two chief constables: As soon as it was known that ‘Bristol was in the hands of the rioters’, he deposed, Bath was ‘in a state of great excitement’.^13^

These elements—excitement that Bath people felt that the Bristol rioters had the upper hand, their strong opposition to the troops going to Bristol, and their attempts to gather information on the Bristol situation—evidence a strong and emotional connection between themselves and people in Bristol. They appeared to have a psychological stake in their neighbours' fate.

What was the basis and nature of this emotional connection between Bath and Bristol? First of all, there were historically strong social connections between the two cities, which are near neighbours, and only 12 miles apart by road–see Figure 2. Bath and Bristol had connections in terms of work, industry, and travel. People clearly came and went between the two cities, and intercity labouring networks are also revealed in the regularity with which magistrates in Bristol enforced removal orders on paupers claiming relief in Bath and vice versa. Industries within easy reach of workers from both cities included numerous coal mines, brass smelting, textile factories, and paper mills. As a leading centre for health and leisure activities for the wealthy, Bath created a considerable demand for domestic servants, many of whom came from Bristol families. Studies of Bristol poll books demonstrate that more than 100 out‐voters were resident in Bath, the majority of whom were artisans and small traders.

Second, Bristol and Bath were both ‘reform’ cities. At least one organized Bristol trade participated in Bath's huge ‘reform’ demonstration in October 1831. And as one Bath‐based newspaper correspondent put it when news of the Bristol riots first emerged, ‘we hope the report is not true that many artisans and labourers of this city have left their work to go and join the rioters of Bristol’. Workers from one factory, he thought, may indeed have done so, ‘but we believe they are nearly all natives of Bristol’.^14^

The longstanding and multiple social connections between Bath and Bristol were the likely basis of psychological and emotional connections. The common identity around ‘reform’ and their common relationship to the anti‐reform authorities and troops likely made salient a sense that at least some of those in Bath saw Bristol people as ‘us’. This would help make sense of the way the Bath crowd took forceful action against the troops, which was clearly intended to delay them getting to Bristol.

This intention was evident in a number of the recorded cries to that effect, as well as their observed actions. Thus the mayor's officer Tothill saw the North Somerset Yeomanry's quartermaster, Bence, ride by in full regimental uniform, pursued by a crowd and take refuge in the Greyhound coaching inn (almost opposite the Guildhall).^15^ Tothill went in to investigate and was joined there by Hall and at least one other mayor's officer, summoned by the landlord because the crowd had stopped outside and was shouting for Bence to leave and not go to Bristol.^16^ Captain Wilkins, distinctive in his Regimentals, was heading towards the White Hart coaching inn, the headquarters of the North Somerset Yeomanry when in Bath, when he was impeded by a crowd of ‘low characters’ who repeated the demand that the yeomanry should not go to Bristol.^17^ They formed a line and forced him back down Stall Street.^18^ He was:

…followed by a mob of several hundred persons, hooting, shouting, and throwing oyster shells and mud at him. The Captain repeatedly attempted to turn his horse towards the mob, but without effect, and he soon cantered down the street.^19^

Wilkins took another route and entered the White Hart by a rear entrance. Protected by the mayor's officers he then addressed the crowd from the front door, saying he was a reformer, but had to do his duty. Members of the crowd responded by ‘crying out, “Out with him, out with the bugger; he shall not go to Bristol”, and shoving with all force to gain an entrance to the inn’. After having removed his distinctive uniform, Wilkins slipped away from the Inn.^20^ Between 6.30 pm and 7.00 pm, Hall and others repeatedly told the crowd that Wilkins had left the White Hart by a rear door, ‘but to no purpose’. For some time, the crowd pushed at the front door while Hall, the Inn's servants, and a handful of others pushed back. Eventually, the front doors were closed, bolted and barricaded, and the crowd responded by attacking the doors, windows, and shutters and, once access was obtained, furniture, with stones, ‘bludgeons, faggot sticks and pieces of broken furniture’.^21^

As the destruction at the White Hart continued, ‘considerable parties’ of rioters broke off to throw stones at the Guildhall windows only 150 m away. The *Bath and Cheltenham Gazette* later speculated that this was to keep the ‘police officers’ occupied in defending the Guildhall so they could not intervene at the White Hart. Whether this was by design or chance it had that effect, so for a period the rioters at the inn were ‘unmolested by any constables or other force’.^22^

The Inn's office was broken into. Inn servant, Daniel Rees, later claimed that he told them, ‘you can't want anything in here, don't destroy the office’, but they replied, ‘we will have the office down and the house too… this is our time; go it, go it my lads. We will serve them as they have at Bristol’.

By the time the attack on the Inn was over, some 487 panes of glass had allegedly been broken and there was structural damage to walls, roofs, ceilings, and floors.^23^ In the estimation of one of the chief constables, there were 300–400 people present by this time. Hall claimed the crowd was much larger – up to 2000 people around the White Hart alone.^24^

After the attack on the White Hart, Hall encountered a crowd in Broad Street, ‘hallooing, shouting and screeching’, and confronted them with some constables. There was hand‐to‐hand fighting, and as Hall later told the assize court, the crowd were ‘drawn up in a regular line like a regiment of soldiers’. They were armed with bludgeons, he said, ‘with which they dared and menaced the constables’. To impede the constables further, aid stone throwing, and perhaps to mask the identity of individuals in the crowd, small boys were hoisted up to the gas lamps in several of these streets to knock them out or to pull down the gas pipes.^25^

Rioting continued in the streets around the Guildhall until about 2.00 am.^26^ Despite the intervention of several hundred special constables, the crowd succeeded in preventing an entire military unit from mustering and delayed its arrival in Bristol until the following day. By the time the Bath troop did arrive in Bristol, the riots there were more or less over.

### The Newport disturbance^27^

On 30th October, as the Bristol rioters turned their attention to the Bishop's Palace after setting fire to the gaols, the mayor rapidly penned a letter to the commanding officer of troops at Cardiff, urging him to send his soldiers to Bristol. The seriousness of the situation in Bristol became apparent to the majority of the population of Newport as darkness fell on the Sunday evening. A reporter for the *Merlin* described the scene:

The utmost anxiety and consternation were depicted on the countenances of the inhabitants of this town on Monday, as they stood in groups about the streets, regarding the fate of the ancient city of Bristol, with which many of them are in some way or other connected. The flames were distinctly seen on Sunday night from the bridge and the churchyard for several hours. At times the horizon was so bright that the reflection upon the Channel was apparently to the extent of half the width of it. The fire was also seen upon the hills beyond Risca.^28^

What was the nature of this ‘connection’ and why were Newport people so concerned about the fate of Bristol? As in the case of Bath, there were long‐standing social and industrial connections between the two. The geographic proximity of Bristol and Newport—~26 miles (see Figure 2)—led to trading links which were developed over several centuries. These encouraged not only the reciprocal movement of commodities across the Bristol Channel, but also the labour required to transport them. By 1825, the number of people with Welsh surnames in Bristol was around 10 per cent of the population, and despite other new arrivals the Welsh retained their status as the largest ethnic minority in the city.

Like Bristol and Bath, Newport was also known as a ‘reform’ town. In relation to the anti‐reform authorities, many Newport people would have therefore seen the pro‐reform Bristol rioters as the same group as themselves.

In addition to these social connections, however, dramatic events nearby likely provided a further framing for Newport people's perception of the authorities' actions in Bristol in October. Just over 3 months before the reform riots, the ‘Merthyr rising’ took place. Using the generalized rallying cry of ‘reform’, Ironworkers repossessed goods seized by the Court of Requests, and then persuaded miners to join them. It took a week for the troops to regain control of the town, actions which left over 20 protesters dead (Williams, 1978). At the time of the reform riots, therefore, Newport people would have been very conscious of the threat to life posed by the troops.

Early on the morning of Monday 31 October at Cardiff Castle, Lieutenant Colonel Love acquiesced to the Bristol mayor's request by mustering 167 men for the relief of Bristol from the three reserve companies of the 11th Regiment of Foot (infantry).^29^ Newport, unlike Cardiff, had a regular steam packet service to Bristol, which offered the means, by requisition, to transport his men in one group.^30^ Love therefore decided to force‐march his unit the 12 miles to Newport.

The troops entered Newport around midday on a working Monday. The soldiers marching in formation in their distinctive red uniforms would have been audible and visible to those people on the street and in adjacent houses, if not further afield. Entering from the south, the troops passed through some of the most deprived parts of Newport, close to the wharves and warehouses of the dock facilities.

The steam packet wharf was on the opposite bank of the River Usk, so Love made directly for Newport Bridge. In his report to the Home Office a few days later, Love stated:

I regret to inform your Lordship that a very bad feeling was shown by the lower orders at Newport on Monday last when I embarked with the Reserve of the 11th Regiment for Bristol; and it was only by taking Military possession of the steamboat that I prevented the mob from cutting her adrift.^31^

This relatively simple (under)statement carries some important information: that a crowd (a ‘mob’) had gathered, and it was composed of the ‘lower orders’ who exhibited ‘a very bad feeling’. It is likely that a crowd gathered in this way only because of foreknowledge of the arrival of the troops or, more spontaneously, when the troops actually marched into Newport. Previous experience of troop movements, the relatively large numbers involved in this case, along with widespread visual and verbal knowledge of the rioting and burning in Bristol the previous night, made the appearance of the 11th Foot logical. By inference, it would have become obvious very rapidly to most people in Newport *where* the troops were going and, with reference to recent events in Merthyr, *what* their role was going to be. It would not have taken long for a crowd of the ‘lower orders’ to deduce that the steam packet moored in full view on the opposite bank of the river was the troop's means of travel.

A contemporary source which adds detail to this event and perhaps had greater access to military sources, comes from an article in the *United Service Journal* of 1831. The Journal contains numerous pieces clearly written by serving officers, though often anonymously. In an article entitled ‘The Riots at Bristol’, the author claimed:

Marching first to Newport, he [Lt. Col. Love] there seized a steamer, which the mob of that place, in complete sympathy with their brethren at Bristol, violently attempted to prevent his occupying. Having prepared a regular attack upon the troops, they were only deterred from carrying it into execution by a few significant words and preparatives on the part of the Commanding Officer, boding a warm reception from the soldiers. Having vainly attempted to cut the boat adrift, their fury found vent in execrations and wishes for the sinking of the vessel ere her crew should trouble their confederates at Bristol.^32^

This article supposes more than just the ‘taking possession’ of the steam packet by the 11th Foot as recounted by Love (above). Instead, we have a confrontation between a crowd there to provide practical support to their fellows (‘complete sympathy with their brethren in Bristol’) and a military unit, with the former ‘having prepared a regular attack upon the troops’. It also suggests that Love threatened the crowd (‘a few significant words’) and that he ordered the soldiers to get ready for violent action (‘preparatives, boding a warm reception’).^33^ His actions were apparently successful.

According to most sources, the men of the 11th Foot eventually marched into central Bristol at 6.00 pm.^34^ Despite the triumphal nature of this entrance, by the time they arrived in the city, the riot had been violently crushed by Dragoon Guards and was effectively over.

Consequently, less than 4 days after their arrival in Bristol and due to fears of further unrest in Merthyr and its environs, on Friday 4 November, Lt. Col. Love and the troops were on the move again, returning to Cardiff via Newport.^35^ The *Cambrian* newspaper noted somewhat quizzically:

We are concerned to hear that the 11th Regiment of Foot was hooted and hissed at Newport … when returning from Bristol … While this highly disgraceful manifestation emanated, no doubt, from the most low and ignorant of the inhabitants, it behoves every well‐wisher of this country to discountenance such proceedings.^36^

This behaviour was in keeping with the confrontation on Monday 31 October, and by this point news concerning the ‘massacre’ of ‘rioters’ in Bristol by British Army units would have been widespread.

### Evidence for process

The Bath and Newport events came closely after collective violence had developed in nearby Bristol. However, the evidence in the form of the actions and utterances of the rioting crowds in each location does not fit well with the idea of ‘contagion’ (e.g., Le Bon, 1965). The nature of the targets for the crowds' violence (the Yeomanry and their headquarters, properties linked to the authorities, special constables), as well as what they left untouched (private homes and shops, for example), reinforces a point made over 40 years ago in social psychology: collective behaviour in even the most violent crowd does not spread uncritically but rather is limited by the definition of social identity shared by participants (Reicher, 1984). These were pro‐reform crowds, and their targets were the forces supporting ‘old corruption’.

This evidence also does not appear to fit the notion that what shaped behaviour was costs‐benefits analysis, in terms of either personal or local interests (e.g., Aidt et al., 2022; Myers, 1997). The crowd in Bath certainly had the power to go further than oppose the Yeomanry's departure for Bristol, yet there is no evidence of looting or other acts of personal gain, and there was no obvious local benefit from confronting the troops. The only buildings targeted and damaged were the White Hart where Wilkins had taken refuge and the Guildhall, where constables were being mustered and captured rioters confined. In Newport, given the context of the recent ‘Merthyr rising’, we might expect the stationing of British troops in south Wales to be contentious, in which case it would seem strange that a crowd would attempt to impede a military unit from *leaving* Wales. We also considered the possibility of a specific recent grievance between residents of both locales and the yeomanry/military units which could have led to the confrontations. Yet outside of a growing perception in many parts of Britain that the Yeomanry were the allies of the anti‐reforming clergy and gentry,^37^ there appeared to be no evidence of such specific local grievances in either location. (Indeed, as manager of a local woollen mill, the part‐time commander of the Bath Yeomanry, Wilkins appears to have been a relatively popular employer).

In Bath, therefore, the violence of the crowd was clearly for some other purpose than personal gain or local benefit. The pattern of evidence—both actions and utterances—suggests this purpose was solidarity with the ‘reform’ rioters in Bristol. In Newport, the confrontation and struggle appeared to have the same purpose. In this case, while we have no recordings of what people in the crowd said, there are several statements from witnesses testifying to the crowd's intentions (to sabotage the journey of the troops to Bristol). Rather than standing to benefit personally or even locally from these actions, participants in fact put themselves at risk of bullet and bayonet wounds, imprisonment, transportation, and even the gallows for people in Bristol.

The evidence of psychological and emotional connections between each of Bath and Newport with Bristol is consistent with the idea that the basis of this solidarity was common identity between participants across the different cities. People in Bath and Newport likely saw the Bristol rioters as the same group as themselves particularly in relation to the anti‐reform authorities and troops. Common identity between riot locations has been documented in some previous research as a mechanism of spread (Drury et al., 2020). However, the events in Bath and Newport were different in an important way from these previously documented cases. In South London in 2011, the motives of rioters who shared identity with those in North London were revenge and retaliation against the police (Drury et al., 2020). In both Bath and Newport, however, while there was also some evidence of retributive motives (‘We will serve them as they have at Bristol’.^38^), the main purpose of the crowd's actions was rather to *prevent* the state forces from using violence against Bristol people in the immediate future. Attempting to block the passage of the troops and attempting to sabotage the boat were not symbolic or even retaliatory acts. We therefore argue that in these case studies we have documented for the first time the existence of *pre‐emptive* solidarity riots. The significance of this for the study of leaderless collective actions is that it evidences that participants in the different locations did indeed see themselves as part of a politically conscious social movement that was bigger than the campaign in their own town or city (Myers & Przybysz, 2010).

## DISCUSSION

The present study has entailed a novel combination of historiographical methods and social psychological analytic concepts—a new kind of collaborative social psycho‐history. Using archives and thick description, and without access to the usual sources for studying collective action (interviews, questionnaires, social media posts, videos), we have nevertheless been able to describe in considerable detail the contours of behaviour in a series of collective action events. Within this, we were also able to make analytic claims about participants' beliefs, perceptions, and motives that are relevant for helping to suggest processes of social influence between some of the events. While we are by no means the first to bring together the two disciplines of psychology and history (e.g., Taine, 1876), and critics on both sides have warned of the dangers of reductionism of such an enterprise (Tileagă & Byford, 2014), this study has been the first to apply this approach to the question of riot diffusion. It has made a case that materials, in particular archive data, and long past events, which are the core focus of historical research, can be brought within the scope of social psychology research.

This combination of historiographical research methods and social psychological concepts has enabled us to make a number of suggestions about the diffusion of collective action events. First, our survey of the 1831 ‘reform’ riots suggested the operation of both independent and interdependent types of collective action event in a single wave. Second, our case studies of the Bath and Newport riots found evidence from multiple sources that these events were responses to the riot in Bristol, not as a form of ‘contagion’ or rational choice based on learning, but as pre‐emptive solidarity. As Hirsch (1990) illustrated so cogently in a study of a student occupation, explanations of collective action in terms of personal cost–benefit analysis cannot explain self‐sacrifice for the cause (except perhaps in a vacuous or circular way). Participants in the Bath and Newport events took personal risks (of death, injury, prison, or transportation) for no observable benefit either to themselves personally or to their local area. And they did so to prevent future action by the authorities, not to punish the authorities for past action (cf. Drury et al., 2020).

It's possible that these solidarity actions were driven by allyship between people who saw themselves as different groups. However, the evidence that there were in fact close bonds between people in the different locations (e.g., ‘brethren’), the emotional links between them, and their identities as pro‐reform cities with a common relationship with the anti‐reform authorities is consistent with the suggestion that there was a common identity between at least some of the participants in each location, and that this was the basis of the action in support of the Bristol rioters, in line with the social identity model (Drury et al., 2020).

As well as contributing to discussion on the nature and diffusion of crowd events (Drury et al., 2020; Reicher, 1984; Stott et al., 2018) the present study is relevant to the broader literature on collective action by pointing towards an area that could be developed in future research. Considerable advances have recently been made in the study of how leaders influence collective action (e.g., Durrheim & Blackwood, 2024; Haslam et al., 2023; Jurstakova et al., 2024; Khumalo et al., 2022). However, most of the main models of collective action neglect the role of norms (González, 2024), and none of them mention influence by observed example. Yet social influence—specifically, the example of others' actions in other collective action events—is a huge factor in collective action participation (Myers, 1997) and one that merits more attention in social psychology.

### Strengths and limitations

Two case studies alone cannot demonstrate that the psychological mechanism of spread elsewhere in the 1831 riot wave, or indeed in other waves, was solidarity based on common identity. Moreover, the fact that we found evidence of solidarity based on common identity doesn't show that other motives were not operating alongside them in Bath and Newport. Indeed, these were not our aims. Our aim was simply to explore whether and how social influence occurred between these events, given the lack of any previous empirical research looking closely at participants' beliefs, perceptions, and motives. The evidence that the two riots were shaped by a solidarity motive suggests, at the very least, that it is problematic to assume that emulation based on cost–benefit analysis is the only or main mechanism of diffusion.

An argument in recent social psychological analyses of riot spread is that local group processes are crucial (Drury et al., 2020, 2022). A key reason why people decide to come onto the streets when collective actions happen elsewhere is often the fact that it is perceived as normative for their peers or local network. By its nature, evidence of meta‐perceptions is less likely to be available to an archive study like the present one than to a study involving interviews.

A question arises over possible differences and similarities of riots in the early nineteenth century with modern urban riots. On the one hand, the belief that defeat or weakness in the police (or troops) in one location could limit their ability to repress a crowd in another location is also a potential feature of modern riots in proximal locations (e.g., Brixton and Clapham in 2011; Drury et al., 2020). On the other hand, the 1831 events, unlike those of 2011, were linked by a nationwide cause (around reform), which may have made pre‐emptive solidarity more likely than in the case of modern urban riots. Arguably, however, this difference is not (only) one of historical features but of types of collective action. One can imagine, for example, striking miners in 1984 believing that a large mobilization in one village could tie up police, thereby limiting their effectiveness in another town.

### Conclusions

Our survey of the 1831 riots has shown how beliefs, perceptions, and motives can be analysed in archive data in a collective action event that would normally be considered outside the scope of social psychology. Our two case studies suggested that the rational‐choice model is not able to explain at least some of the patterns of influence between locations. Instead, the evidence of pre‐emptive solidarity could be explained by suggesting there was a common identity between Bristol and each of Bath and Newport. The present study therefore demonstrates the value of historiographical research methods for social psychology and how psychology can be informed by incorporating techniques from other disciplines that capture human action, as well as contributing to a richer, more psychologically informed approach to studying the crowd in historical research.

## AUTHOR CONTRIBUTIONS

**John Drury:** Conceptualization; funding acquisition; writing – original draft; writing – review and editing. **Roger Ball:** Conceptualization; investigation; funding acquisition; writing – review and editing; formal analysis; methodology; data curation. **Steve Poole:** Conceptualization; investigation; funding acquisition; writing – review and editing; formal analysis; supervision.

## FUNDING INFORMATION

Economic and Social Research Council. Grant number ES/T00293X/1.

## CONFLICT OF INTEREST STATEMENT

None of the authors have a conflict of interest to disclose.

## Data Availability

All data used in this paper are sourced from publicly available archives: newspapers (Times Digital Archive, ProQuest, British Newspaper Archive, National Library of Wales, Somerset Heritage Centre, Bath Records Office); correspondence and court papers (The National Archives, Bath Record Office, Newport Museum and Art Gallery Local Studies collection, Somerset Heritage Centre); contemporary governmental reports (online); contemporary regimental histories of Yeomanry units; Victoria Art Gallery (contemporary images). Additionally, we drew on a dataset on locations of Political Unions given in LoPatin (1999).

## Footnotes

^1^
Our use of the term ‘psycho‐history’ is a deliberate reference to the approach of the early crowd psychologist Taine (1876). What we have in common with that work is the ambition to enrich historical studies with psychology. A fundamental difference between our two approaches is that we draw upon concepts from modern social psychology and combine them with historical scholarship, whereas Taine's reductionist approach treated (selective) historical events as simple effects of a primitive collective psychology (see Stott & Drury, 2017).

^2^
Further details of the categories of information gathered can be found in Ball et al. (2021).

^3^
The ‘late modern period’ covers the years 1800–1945.

^4^
This was only possible for the Bath riot as in the Newport incident there were apparently no arrests.

^5^
This summary is based on more comprehensive case studies of the Bristol riot by Poole  (2023a, 2023b) and Poole and Ball (forthcoming).

^6^
‘Special constables’ were volunteers who signed up for a limited period to assist regular constables and magistrates typically for public order situations.

^7^
Parts of the narrative are based on a more comprehensive case study by Poole et al. (2024).

^8^
The National Archives (TNA) HO 52/15, f.597 Copy of letter from JPs Charles Cook and William Clarke to Captain Charles Wilkins 30 October 1831.

^9^
TNA, HO 52/15, ff.593–594 & 596, Marquess of Bath to Melbourne, enclosing Captain Wilkins's explanation of events, 4 and 14 November 1831.

^10^
Bath Record Office (BRO), Philip George Papers, bundle 206, information of William Hall, 16 November 1831.

^11^
TNA HO 52/15, f.624, Kitson to Melbourne, 31 October 1831; *London Courier* 1 November 1831. According to the Courier's Bath correspondent, the Bristol fires could be seen from both the Old Bridge and Norfolk Crescent, and ‘the horizon illuminated with almost equal grandeur as the setting sun’.

^12^

*Sherborne Mercury* 9 April 1832.

^13^
BRO, Philip George Papers, bundle 206, information of Chief Constable, 16 November 1831.

^14^

*London Courier* 1 November 1831.

^15^
The Guildhall was Bath's administrative centre, the seat of the Corporation and magistrates, and the site of its law courts.

^16^
BRO, Philip George Papers, bundle 206, information of Robert Tothill, 16 November 1831.

^17^

*Bath Herald* 5 November 1831.

^18^
BRO, Philip George Papers, bundle 206, information of William Hall (second testimony, not dated).

^19^

*Bath and Cheltenham Gazette* 3 April 1832.

^20^

*Bath Chronicle*, 3 November 1831; *Bath and Cheltenham Gazette* 1 November 1831; *Bath Herald* 5 November 1831.

^21^
Information of William Hall (second testimony, not dated); prosecution brief vs. John Holbein and John Durnell; *Bath and Cheltenham Gazette* 3 April 1832.

^22^

*Bath and Cheltenham Gazette* 1 November 1831; *Bath Herald* 5 November 1831.

^23^

*Bath Chronicle* 5 April 1831; information of William Sims, 31 October 1831.

^24^
BRO, Philip George Papers, bundle 206, First information of William Hall, 31 October 1831.

^25^
Information of William Hall (first testimony, 31 October 1831 and second testimony, not dated); prosecution brief vs. Benjamin Stride, John Macey and Joseph Maggs; *Bath Chronicle* 5 April 1832.

^26^

*Sherborne Mercury* 9 April 1831.

^27^
This analysis is based on a more comprehensive case study by Ball (2024).

^28^

*Monmouthshire Merlin* 5 November 1831. This was confirmed by *Bath Chronicle* 10 November 1831.

^29^
TNA HO 40/28/01 Home Office: Disturbances Correspondence‐Activities in distressed areas Civil and military reports‐Bristol, riots–Col. Love (OC Cardiff) to Lord Melbourne ff.24–25; TNA WO12/2860 Commissary General of Musters Office and successors: General Muster Books and Pay Lists–Infantry‐11th Foot muster rolls 1831.

^30^

*Monmouthshire Merlin* 29 October 1831.

^31^
TNA HO 52/16 Counties Correspondence: Warwick‐York, Wales and miscellaneous–Lt. Col. Love to Lord Melbourne ff.138–140.

^32^
“The Riots at Bristol” *The United Service Journal* Vol. III (London: Samuel Bentley, 1831) p. 439.

^33^
Preparing a ‘a warm reception’ is a military term for getting ready to use violence against an advancing opponent. In this case it could refer to a unit loading rifles with ball (shot) or fixing bayonets.

^34^
Amey, *City Under Fire* p.106; Eagles, J, *The Bristol riots, their causes, progress, and consequences* (1832); *The Bristol Job Nott, or Labouring Man's Friend, issue VI* 19 January 1832 p. 22.

^35^
TNA HO 41/10 Home Office: Disturbances Entry Books‐1831 April‐Dec 31 Oct 1831 Lamb to J. B. Bruce ff. 160–161; TNA WO12/2860 Commissary General of Musters Office and successors: General Muster Books and Pay Lists–Infantry‐11th Foot muster rolls 1831; *Monmouthshire Merlin* 12 November 1831.

^36^

*The Cambrian* states this event occurred on ‘Monday last’ (7 November) but this appears to be in error, all the other evidence suggests Friday 4 November. *The Cambrian* 12 November 1831.

^37^
These issues are discussed in more detail in Ball, Poole, and Drury (2023a; pp. 8–9, 10–14.

^38^
The slang term ‘to serve them’ means to punish.
